# Comparison of EOG and VOG obtained eye movements during horizontal head impulse testing

**DOI:** 10.3389/fneur.2022.917413

**Published:** 2022-09-02

**Authors:** Maksim Pleshkov, Vasilii Zaitsev, Dmitrii Starkov, Vladimir Demkin, Herman Kingma, Raymond van de Berg

**Affiliations:** ^1^Department of Otorhinolaryngology and Head and Neck Surgery, Division of Balance Disorders, Maastricht University Medical Center, School for Mental Health and Neuroscience, Maastricht, Netherlands; ^2^Faculty of Physics, National Research Tomsk State University, Tomsk, Russia

**Keywords:** electro-oculography, video-oculography, head impulse test, eye velocity, asymmetry

## Abstract

**Introduction:**

Video head impulse testing is frequently used to evaluate the vestibular function. During this test, eye movement responses are recorded with video-oculography (VOG). However, the use of VOG can sometimes be challenging, especially due to pupil detection problems (e.g., blinking, droopy eyelids, etc.). Therefore, this study investigated whether electro-oculography (EOG), a technique that does not depend on pupil tracking but on the orientation of the corneoretinal potential, might be an alternative to VOG for quantifying eye movement responses during head impulse testing.

**Subjects and methods:**

Head impulse testing was performed in 19 healthy subjects without a prior history of vestibular symptoms. Horizontal eye movements were recorded simultaneously with EOG (using an EOG system) and VOG (using a VHIT system: ICS Impulse). The eye movement responses to each side of both techniques were compared using a concordance correlation coefficient (*r*_*c*_), *t*-testing, and Bayes Factor (BF) paired *t*-testing.

**Results:**

EOG and VOG obtained eye movement traces that correlated well with each other during head impulse testing (average *r*_*c*_ = 0.89). Average VOR gains obtained with EOG and VOG were not significantly different from each other for all subjects during left head impulses. However, VOG gains differed between both techniques regarding right head impulses. VOG showed significant VOR gain asymmetry (5% to the right), whereas EOG showed no significant asymmetry (1% to the right).

**Conclusion:**

This study demonstrated the use of EOG to record eye movements during head impulse testing for the first time. EOG and VOG obtained eye movement traces that correlated well with each other during horizontal head impulse testing. In addition, EOG showed smaller VOR gain asymmetry in healthy individuals, in contrast to VOG. These findings indicate that EOG might potentially be applicable as an alternative to VOG for collecting eye movement responses during head impulse testing.

**Trial registration number:**

10192021-38 dated 19.10.21.

## Introduction

The vestibular organ is a part of the inner ear, located in the temporal bone on both sides of the head (1). Each organ consists of three semicircular canals sensitive to head angular accelerations and two otolith organs sensitive to head linear accelerations and gravity (2).

One of the semicircular canals functions is to stabilize images on the retina during head movements. This is established by the vestibulo-ocular reflex (VOR), which moves the eyes, velocity controlled, in the opposite direction of the head movement (3). For example, when the head rotates to the right, the eyes rotate to the left, keeping the image of a tracked object almost stationary on the retina. However, various diseases and injuries of the inner ear can lead to an impairment of the vestibular function (4), leading to a reduced VOR (5).

A frequently used test to assess vestibular function is the head impulse test (HIT) (6). During HIT, the examiner performs abrupt, unpredictable, fast, and small amplitude rotational movements of the patient's head (also called head impulses), while the patient tries to fixate on an earth-fixed target placed in front of the patient. During the head impulse, the eye movements are observed and analyzed. If the VOR is intact, eye and head velocities will be in opposite direction but of the same magnitude, resulting in a smooth eye movement without interruptions. However, if the VOR is impaired, the patient's eyes will not move or move too slowly, and the visual fixation of the target will be lost. In order to regain target fixation, corrective saccades will be made either during (covert saccade) or after (overt saccades) the head impulse to bring the eyes back on target. The HIT can evaluate the VOR in all three dimensions and investigate the function of all six semicircular canals (6).

Various eye-tracking methods have been developed to quantify the VOR (7, 8). Electro-oculography (EOG) and video-oculography (VOG) are the most commonly used techniques in clinical practice. EOG is a low-cost technique, already used for decades, based on the fact that the eye can be considered as a dipole since the electrical activity in the retina leads to a corneoretinal potential. During the eye movements, the orientation of this dipole changes, which can be detected by electrodes placed around the eyes (9–11). VOG is a more recently developed technique based on pupil detection, using an infrared video camera that is (in most devices) mounted on relatively expensive goggles (12–14).

In order to quantify the VOR during HIT, the video head impulse test (VHIT) was developed using low-weight goggles with a built-in motion sensor and a high-speed infrared camera. These commercially available devices can track eye and head movements during HIT (12–15). The recorded eye and head velocities are then analyzed to quantify the VOR using the most crucial outcome parameter “gain”: the relation between eye and head velocities during HIT (15). The VHIT also identifies corrective covert and overt saccades that compensate for the defective VOR. Currently, most VHIT devices use one infrared camera (VOG) mounted on the goggles, which tracks eye movements of one eye. Unfortunately, during the fast head impulses with peak velocities between 200 and 300 deg/s, the mass inertia of the goggles can induce substantial artifacts due to slippage of the goggles over the head, despite the minimal weight and very tight strapping (16, 17). The accuracy of the VHIT relies also on an accurate pupil position detection, which can be hampered by eye blinking, drooping eyelids, and eyelids covering the pupil (e.g., narrow eyelids or big pupils) and other issues. The sample frequencies of the VHIT systems are currently limited to 250 Hz, and the camera position (right or left eye) has been suggested to induce a non-physiological asymmetry in VOR gain (18). Although VHIT is a clinically highly relevant and frequently used vestibular function test, it is no “plug and play.” It requires substantial expertise and training to manually perform the correct head impulses and to recognize and deal with problems of eye-tracking and goggle slippage artifacts. Commercially available VHIT devices are still relatively expensive due to their special goggle design, hardware, and software.

The advantage of EOG over VOG is that it works in almost any subject, even with eyes partially or completely closed. Eye blinks are one of the main sources of artifact with EOG, like in VOG. EOG is also a less expensive but very robust technique, using electrodes (either disposable or reusable) and a simple multichannel differential amplifier able to detect horizontal and vertical eye movements at high sampling rates, especially also for fast eye movements like saccades (19).

However, EOG is not a “plug and play” technique. It requires special expertise and training to mount the electrodes correctly in order to minimize the signal-to-noise ratio and drift. Furthermore, the light intensity in the examination room needs to be kept constant, because the corneoretinal potential directly depends on the intensity of the incident light on the retina. These aspects require a regular calibration of the corneoretinal potential as a measure of the eye rotation. To obtain an optimal signal-to-noise ratio, a bright minimum of 200 lux illumination for the HIT experiments is used and recommended. Physical contact with the electrodes or movement of the skin has to be prevented when using EOG for HIT, as they induce severe artifacts (similar to goggle movements with VOG).

This study aims to check the feasibility of using EOG as an eye movement detection technique during horizontal HIT, by comparing eye movement responses detected with EOG to those simultaneously obtained with VOG. Secondly, it investigates whether the gain asymmetry for leftward and rightward head impulses as has been reported when using VOG in healthy individuals can be confirmed when detecting the eye movements with EOG.

## Methods and materials

### Study design

HIT was performed in 19 healthy subjects, while eye movements were recorded simultaneously with EOG (using an EOG system) and VOG (using a VHIT system). Horizontal eye movements were compared between EOG and VOG using concordance correlation and Bayes factor analysis as well as *t*-testing.

### Study population

Nineteen healthy subjects without a prior history of vestibular symptoms were included in this study. A group of subjects included 14 men aged 23.0 ± 3.8 years (mean ± standard deviation) and 9 women aged 24.7 ± 4.1 years (mean ± standard deviation).

### EOG setup and preparation

The EOG system consisted of a custom-made 8-channel differential amplifier with a 20 Bits ADC, ± 50 *mV* measurement range, and 0.1 μV resolution (MPAQ, IDEE, Maastricht University, the Netherlands). The amplifier was connected to the PC *via* a USB interface. Custom-made software (IDEEQ, IDEE, Maastricht University, the Netherlands) was used for signal acquisition and preprocessing. The recorded signal was hardware filtered using a 50-Hz low-pass filter. The signal amplification factor (gain) was set at 3,200.

**Figure 1:**
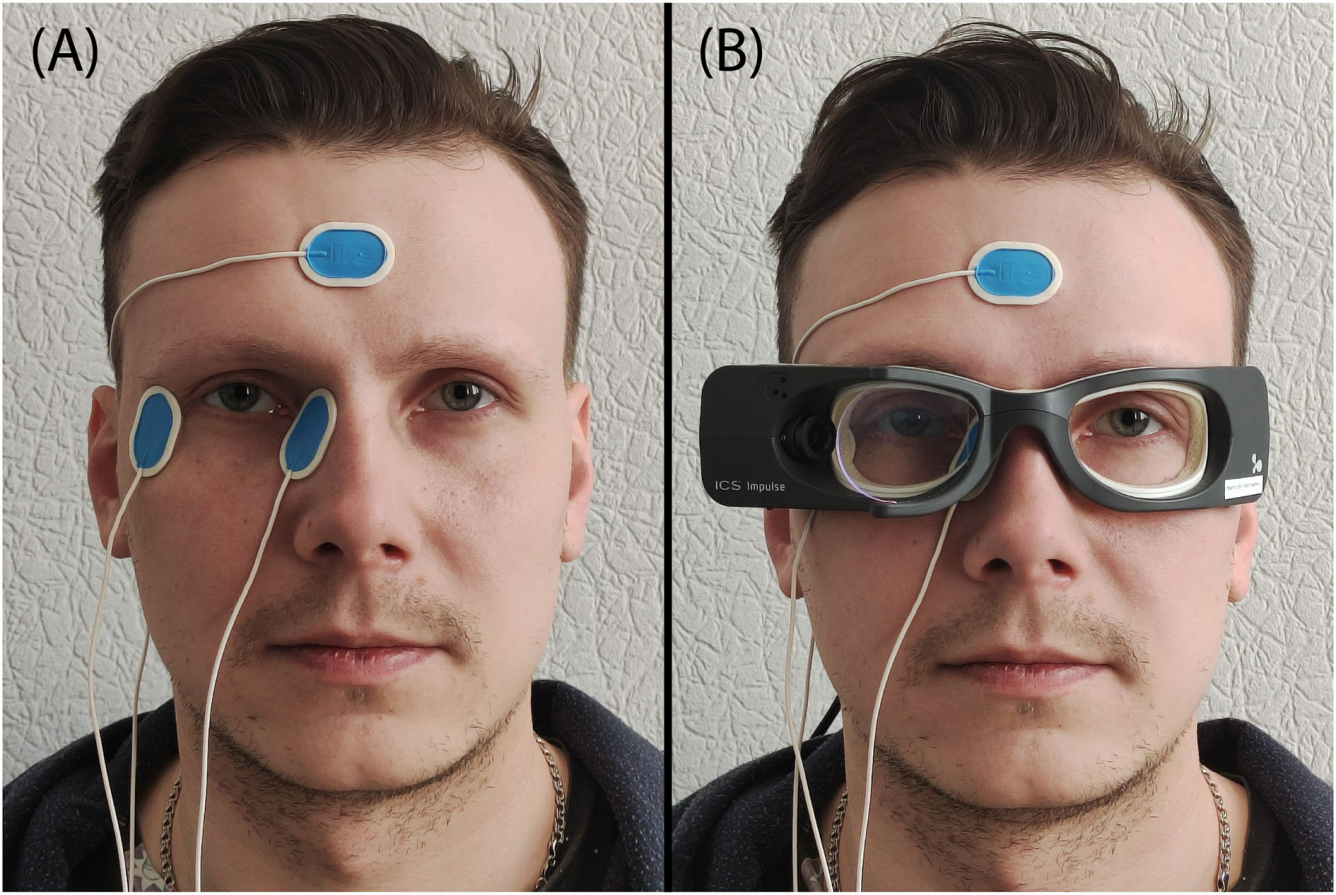
Illustration of the placement of the electrodes and VOG goggles. **(A)** Location of the EOG electrodes, **(B)** ICS Impulse goggles together with the EOG electrodes.

To detect the corneoretinal potential, three disposable Ag/AgCl electrodes (Ambu Blue sensor N-50-K/25, prewired, 30 by 22 mm size, 1.5 mm connector, Ballerup, Denmark) were used. The skin located to the right and left of the right eye, and the skin on each subject's forehead, were cleaned with petroleum ether to optimize the electrical contact and allow proper electrode fixation. Two electrodes were put on the left and right sides of the right eye (monocular, naso-temporal derivation of the eye position). The reference electrode was placed on the subject's forehead (Figure 1A). The EOG sampling rate was set at 250 Hz.

The EOG system was calibrated manually. Three markers were placed along with a horizontal line on a well-illuminated white painted wall (cold white light of fluorescent lamps, 200 lux ± 4%, measured by an RGK LM-20 lux meter) with a fixation distance of 2 m. Light conditions were carefully kept constant to avoid changes in corneoretinal potential. After placing the electrodes, subjects were sitting for 15 min in front of the illuminated wall, to allow the corneoretinal potential to be stabilized. The calibration markers were placed on the wall at a mutual distance of 7.5°. The healthy subject was then asked to look at the markers one by one while the head was kept stable by the examiner. The degree-voltage relation for EOG can be assumed to be linear, especially for the small amplitude of eye movements (10–15°) (20). Therefore, calibration was performed by fitting the linear regression on three data points. The electro-corneal potentials ranged between 0.49 and 1.20 UV per degree of eye rotation for all 19 subjects.

### VHIT setup and preparation

ICS Impulse goggles (GN Otometrics, Taastrup, Denmark) were used for VHIT. This device incorporates a high-speed infrared camera recording the right eye and a set of gyroscopes to detect head movements. To avoid contact, the goggles were put on the subject's face above the already-fixed EOG electrodes (Figure 1B). It was tightly strapped on the subject's head to prevent slippage. The sampling rate was set at default 246 Hz in OtoSuite software (GN Otometrics, Taastrup, Denmark).

For calibration, an automatic built-in horizontal two-point laser calibration was used with a fixation distance of 2 m. The same well-illuminated wall was used for EOG calibration (see above). A detailed description can be found in the ICS Impulse Reference Manual: https://partners.natus.com/asset/resource/file/otometrics/asset/2019-07/7-50-2060-EN_05.PDF.

### HIT testing

Horizontal eye movements during horizontal HIT were recorded by EOG and VOG simultaneously. Subjects were seated on a chair. Subjects then were asked to fixate a stable dark blue target at a distance of 2 m, placed at a height of 1.17 m from the floor on the well-illuminated wall (see above). The head of the subject was flexed for 20° to ensure that the impulses were made approximately in the plane of the horizontal canals and to ensure a good pupil capture by the VOG camera, also at lateral gaze (± 7.5° from the center). One trained examiner (VZ) performed all impulses: small amplitude (10–15°) fast (>120°/s) head impulses in the horizontal plane. These outward unpredictable head impulses always started from the center and were randomly applied to the left and right until the ICS Impulse detected 20 valid impulses to each side.

### Data processing

The data were processed in Matlab 2014b software. First, a 30-Hz software low-pass filter was used to reduce the noise of all traces (21). After that, the VOG velocity trace was additionally filtered with a 20-Hz low-pass filter since it contained a relatively low-frequency (20–30 Hz) noise. For EOG, eye velocities were calculated by the first-order 5-point central difference of the recorded eye position. Both signals were resampled to 245 Hz, using spline interpolation to eliminate sampling time instabilities and allow further point-to-point comparison.

The VOG and EOG eye velocity traces were first synchronized in time by finding the cross-correlation maximum (best-match) between the whole eye velocity traces, whereas VOG eye and head velocity traces were already synchronized by means of the built-in VOG system procedures. The position of every head impulse was identified by determining the timing of head peak velocities. For the analysis, 800 ms of each head impulse test was selected: 200 ms before the head peak velocity and 600 ms after the head peak velocity. Impulses were only included in the data cleaning process, if they were accepted by the VHIT system. Therefore, no artifacts were additionally removed from the EOG velocity traces. For each subject, data cleaning implied that head impulses were included in the analysis if the head peak velocity ranged between 120 and 250 deg/s.

The signal-to-noise ratio (SNR) was calculated for unfiltered, correlated, and length-adjusted EOG and VOG eye velocity traces as $SNR=10^{*}log10\left(\frac{P_{signal}}{P_{noise}}\right)$ [in dB], where *P*_*signal*_ and *P*_*noise*_ are the signal and noise power, respectively. The signal and noise power were calculated as a square of the amplitudes in a frequency domain after performing the Fourier transform. The signal was considered to be present in a range (0, 30] Hz (21), the noise consequently took the rest of the bandwidth [30, 122.5] Hz.

The VOR gains were calculated for every EOG and VOG obtained trace, as the ratio of the areas under the eye velocity and head velocity curves [area under the curve from 40 ms before to 80 ms after peak head velocity as introduced in Synapsis system (22)] and were then averaged per subject.

Asymmetry was calculated again for every included impulse as $asymmetry=\frac{Gain_{Left}-Gain_{Right}}{Gain_{Left}+Gain_{Right}}$, where *Gain*_*Left*_ and *Gain*_*Right*_ comprised gains calculated as described above, during left and right impulses, respectively. This implies that negative asymmetry values indicated an asymmetry to the right, and vice versa. The value of 0 indicated the absence of asymmetry.

It should be noted that for both VOG and EOG the motion sensors in the VOG goggles were used to detect head movements. Hence, in this study, it was not possible to investigate difference in latencies using EOG or VOG (see limitations of this study).

### Data analysis

For every subject, concordance correlation analysis was performed for each impulse and for each side, to estimate the correlation between eye velocity traces recorded by EOG and VOG. On top of that, the concordance correlation analysis was applied for the head velocity traces and VOG and EOG obtained eye velocity traces. The obtained concordance correlation coefficients (*r*_*c*_) were first z-transformed (by Fischer), then averaged per subject, averaged per group, and finally the average *r*_*c*_ was obtained for the whole tested group after the inverse Fisher z-transform. *r*_*c*_ values close to 1 implied perfect concordance, whereas a value of zero indicated the absence of concordance. Bayesian factor (BF) paired *t*-testing was used to compare the average VOR gains obtained by EOG and VOG to each side; *t*-testing was applied to separately compare VOR gain asymmetries of EOG and VOG obtained eye velocity traces with the value of 0 (indicating absence of asymmetry). BF values >1 indicated evidence of an alternative hypothesis (significant difference), values <1 implicated evidence for the null hypothesis (no significant difference), and a value of 1 indicated no evidence for any hypothesis. *T*-test *p*-values lower than 0.05 were considered statistically significant.

## Results

### Comparison of eye velocities obtained with EOG and VOG

Peak head velocity during head impulses ranged from 120.7 to 249.2 deg/s (mean ± standard deviation 174.4±20.4 *deg*/*s*). The SNR for VOG and EOG was equal to 6.1 dB and 3.2 dB, respectively.

**Figure 2:**
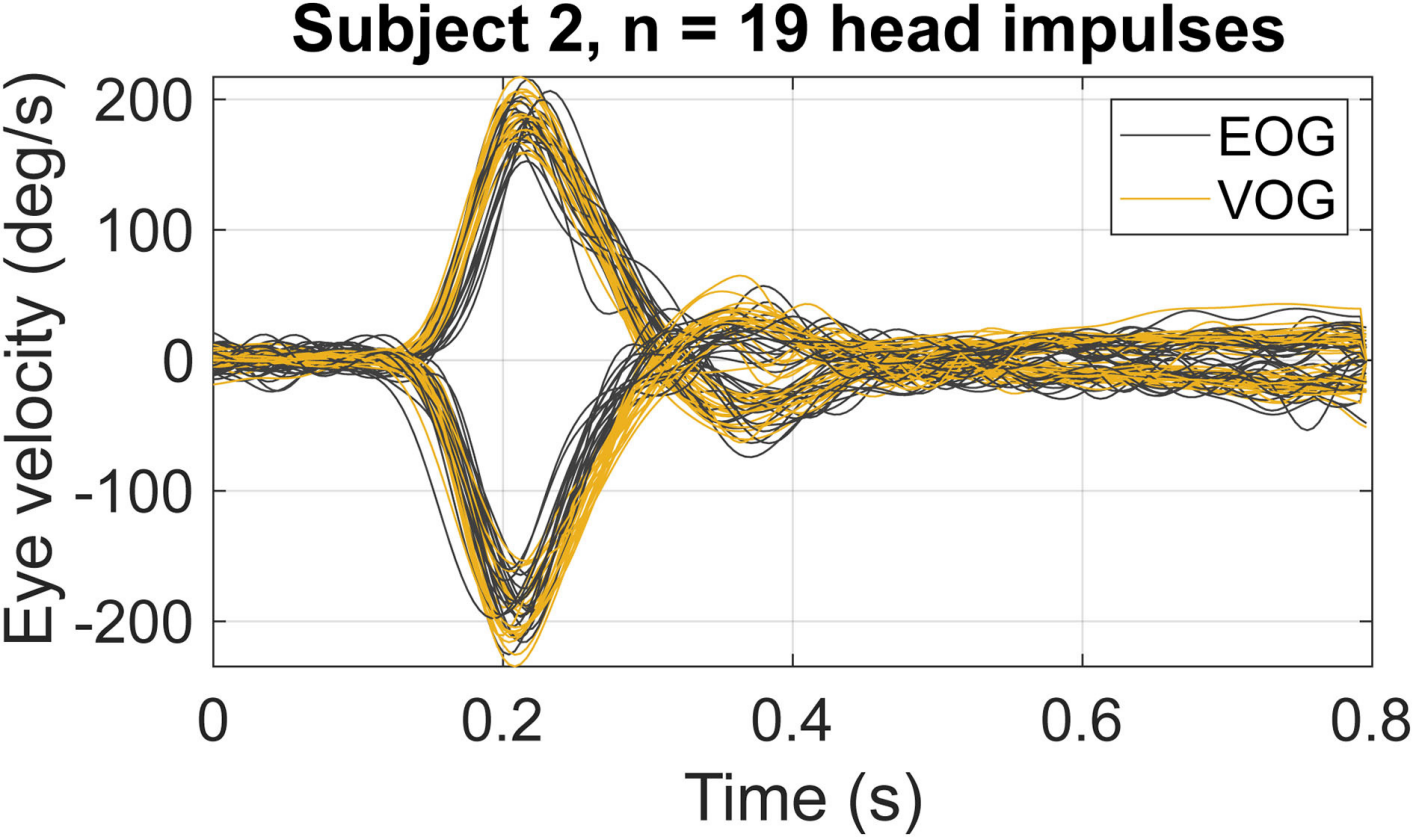
An example of eye velocity traces in a single subject (subject 2), during 19 head impulses to the left (positive velocities) and to the right (negative velocities), recorded by EOG (gray traces) and VOG (yellow traces).

After data cleaning, every subject had at least 19 valid head impulses to each side; therefore, 19 head impulses were used for the analysis. The latency between the peak head velocity and the peak eye velocity detected by VOG in this study was on average 6.6 ms to the right and 8.7 ms to the left. Figure 2 presents an example of the obtained eye velocity traces in a single subject during 19 head impulses to the left and the right, recorded by EOG (gray traces) and VOG (yellow traces).

The average *r*_*c*_ coefficient of all subjects was equal to 0.89, indicating a positive correlation between EOG and VOG eye velocity traces. The average *r*_*c*_ when comparing VOG eye velocity and head velocity traces was 0.96, whereas average *r*_*c*_ for EOG was 0.89.

### Comparison of VOR gain between EOG and VOG

The mean VOR gains for left and right head impulses and their standard deviations for both EOG and VOG are illustrated in Table 1. Using a BF-paired *t*-test, the difference between the average VOR gain for EOG and VOG was not significantly different from zero for all subjects for left head impulses (mean difference = −0.01, df = 18, t = −0.41, *p* = 0.169, BF = 0.26). VOG gains differed between both techniques regarding head impulses to the right (mean difference = −0.08, df = 18, t = −2.32, *p* = 0.03, BF = 2.03).

**Table 1:** Mean VOR gains for left and right impulses and their standard deviations for each oculography system (*n* = 19).

**EOG**		**VOG**	
**Left**	**Right**	**Left**	**Right**
0.93 ± 0.11	0.95 ± 0.17	0.94 ± 0.10	1.04 ± 0.12

### Comparison of VOR gain asymmetry between EOG and VOG

**Figure 3:**
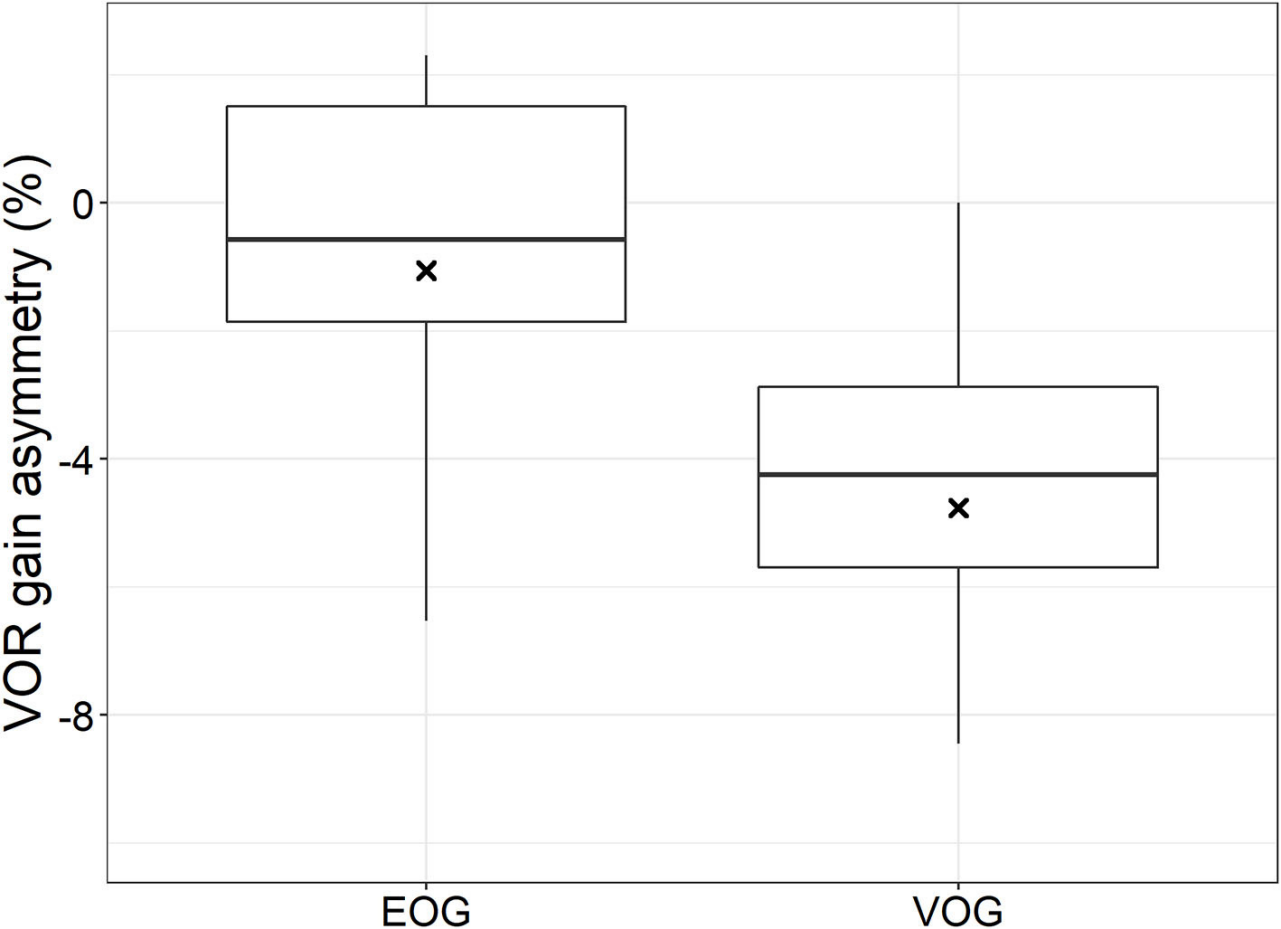
Boxplot: left-right asymmetry in VOR gain calculated for EOG and VOG obtained traces. Black crosses indicate mean values, black bars indicate medians, boxes represent first and third quartiles, and dots represent outliers.

Figure 3 shows the VOR gain asymmetries obtained with EOG and VOG during horizontal HIT for the whole group. The mean VOR gain asymmetry obtained with EOG was 1% to the right, which was not significantly different from 0% asymmetry (*t*-test: mean = −0.01, df = 18, t = −0.49, *p* = 0.62). On the opposite, the mean VOR gain asymmetry obtained with VOG was 5% to the right, which was significantly different from 0% (*t*-test: mean = −0.05, df = 18, t = −7.41, *p* < 0.001). Furthermore, EOG and VOG gain asymmetries were not significantly different from each other (paired *t*-test: mean difference = 0.04, df = 18, t = 1.75, *p* = 0.10). EOG-obtained VOR gain asymmetries were more dispersed than those obtained with VOG (Figure 3).

## Discussion

This study compared eye movement traces of the right eye, which were simultaneously collected using EOG and VOG during horizontal head impulse testing, in a group of 19 healthy subjects. The EOG and VOG obtained eye movement traces correlated well with each other. Nevertheless, the VOG eye velocity correlated better with the head velocity traces than the EOG as compared to concordance correlation coefficients. The obtained SNR in VOG eye velocity traces was 3 dB higher before filtering compared with EOG SNR. The VOG obtained latencies between peak head and peak eye velocities agree with the values reported in the literature for the latency of the VOR (23). The VOR gains were not statistically different between EOG and VOG methods during impulses to the left but significantly differed during impulses to the right. Furthermore, a 1% not significant and a 5% significant VOR gain asymmetries to the right were present in the eye movement traces obtained with EOG and VOG, respectively. These findings indicate that EOG might potentially be applicable as an alternative to VOG for collecting eye movement responses during head impulse testing.

EOG and VOG eye movement responses correlated well, although both techniques do not exactly provide the same type of traces, which is inherent to their different physical backgrounds (24). After all, EOG recordings can be distorted by electrode polarization processes, motion artifacts, power-line interferences, muscle contraction, intrinsic device noise, and other processes (25). These factors probably resulted in greater values of noise (less precision), as can be seen from SNR values assuming the same signal magnitude. Nevertheless, the applied filtering should have equalized the SNR for EOG and VOG, although the levels of low-frequency (<30 Hz) noises remain unknown. VOG recordings, on the other hand, can mainly be distorted by pupil detection problems (e.g., due to small eyelids, blinking, etc.) (26). So far, VOG seems to better detect eye movements in terms of the recorded velocity traces, assuming the (nearly) perfect concordence between head and eye velocities in the case of intact VOR.

Furthermore, the observed correlation between eye movement responses measured with both oculography methods does not imply that the same VOR gains should be obtained with both techniques. Therefore, both the correlation and VOR gain should be considered when investigating the feasibility of EOG as a method for head impulse testing. It can be concluded that EOG reliably detects high-velocity eye movements during horizontal head impulse testing, since (a) this study showed a well correlation between eye velocity traces recorded by both techniques; (b) the VOR gains were very similar between both techniques despite a significant difference for head impulses to the right (not to the left). After all, the significant VOR gain difference of 0.08 is most likely not clinically significant. Although using EOG is more time-consuming than VOG, EOG has several advantages: (1) Pupil detection is not necessary. This suggests that small eyelids, droopy eyelids, lens implants, and testing with lateral gaze (head impulses in the vertical plane with only a little space for the pupil before it disappears behind the eyelids) (27, 28) might not lead to artifacts in the traces. Even mini-blinks might not lead to substantial artifacts, as long as the horizontal EOG trace is not significantly interrupted by the blink; (2) Both eyes can easily be measured by applying electrodes around both eyes (29). Most current VOG systems for head impulse testing only use one camera to record one eye; (3) EOG does not require goggles to measure the eye movements. Therefore, the shape (size) of the head is less important to perform this examination (e.g., small children) (30). Many currently available VOG systems for head impulse testing still use a camera mounted on goggles. Taking all these factors into account, this might imply that EOG could be used as an alternative to, or complementary to, VOG for head impulse testing in the future. Nevertheless, if EOG would be used for head impulse testing, still a solution should be found to measure head movements. After all, in VHIT most often gyroscopes are incorporated in the head-mounted goggles, while EOG does not use head-mounted goggles.

Regarding VOR gain asymmetry, it was found that EOG-obtained eye movement responses showed small but not statistically significant asymmetry of 1% to the right, while those obtained by VOG did demonstrate a significant asymmetry of 5% to the right (which is the side of the camera and the recorded eye). These differences in asymmetry are congruent with the significant differences in VOR gain between EOG and VOG, regarding head impulses to the right. The 5% asymmetry obtained with VOG, with higher VOR gains obtained during head impulses to the side of the camera, corresponds with the literature (18). Several hypotheses were previously proposed to account for a physiological basis of this VOG detected asymmetry, for example: (1) Adduction requires an additional neuron compared to abduction. Therefore, during a head impulse to the right, the adducting (right) eye has a longer latency and needs a higher velocity to catch up with the head movement (18, 31); (2) The axis of head rotation and distance to the fixation target might influence VOR gain in favor of the side of the camera (18, 32). The asymmetries observed in this study together with those reported in the literature (31) suggest that it is not likely the artifact of one of the oculography systems itself. Also, the left-right asymmetry present at relatively low head velocities (~180 deg/s) might be too small (31) to be detected by EOG for instance due to the lower resolution (8) compared with VOG. On top of that, a previously described asymmetry in corneoretinal potential (a lower potential when the eye is turning from right to left) might counterbalance the inherent asymmetry of the oculomotor system (19, 33) leading therefore to lower asymmetry values in the EOG recorded traces using monocular recording.

This study demonstrated that EOG can reliably detect eye movements during head impulses. However, in order to explore whether EOG is feasible for head impulse testing per se, more research is needed. In particular, EOG should be tested during head impulse testing in patients with vestibular pathology, in order to assess whether EOG can accurately detect compensatory saccades which occur in the case of vestibular hypofunction (22). In addition, the EOG technique could be optimized, e.g.,: vertical electrodes could be used to explore the feasibility of vertical head impulse testing with EOG and to detect vertical eye velocities that present in eye blinks. The use of binocular eye recordings with EOG might compensate for a possible VOR gain asymmetry (resulting in less dispersion of VOR gain asymmetries in the EOG recordings, see Figure 3) since the electrodes are not placed symmetrically around the eyes with respect to the optical axis (21).

### Limitations

Main limitation of this study involved data selection: only impulses were used, which were accepted by the vHIT device (i.e., VOG eye movement responses). This implies that a selection bias was present with respect to VOG recordings. After all, if e.g., an eye movement response during head impulse testing was rejected by the vHIT device due to pupil detection failure, this would not necessarily have been a problem for EOG, since it does not depend on pupil detection. Therefore, this study did not facilitate a comparison between EOG and VOG regarding the influence of artifacts on VOR outcomes. Therefore, dedicated new algorithms have to be developed to identify correct impulses appropriate for quantification of vestibular function, before EOG will be used for HIT.

For both eye movement detection techniques, the motion sensors in the VOG goggles were used to detect the head movements. Hence, in this study, goggle slippage might have affected the latency between eye and head movements for both EOG and VOG recordings in the same way. The latency between the peak head velocity and the peak eye velocity detected by VOG in this study was on average 6.6 ms to right and 8.7 ms to the left and falls within the normal range of the VOR latency. The absolute latency of the EOG measurements defined as the time span between the onset of the eye movement and the change in the corneoretinal potential is virtually non-existent, as these phenomena are mechanically coupled. The detection of the corneoretinal potential change depends therefore on the signal processing in the data-acquisition system. The delay in this system is specified as 80 μs. The delay of the eye movement detection in the VOG system is not specified, but as this is based on image processing and a frame rate of 246 Hz, it is longer than 4 ms, which is order of magnitude longer than the delay in the EOG system. The detection of the real onset of the eye movement in both systems is determined by algorithms that depend on the signal-to-noise ratio.

Compared to motion sensors in the goggles, a head-fixed motion sensor mounted on a bite bar (34) might probably provide more accurate information about the head movements, as this ensures close contact with the skull. It is well known that eye movement detection with VOG can be disturbed by the movement of the goggles relative to the eyes (26). Similarly, movement of the electrodes with EOG can disturb the eye movement detection, but most likely in a very different way and should be avoided at all expenses. So recognition of specific artifacts when using EOG for HIT is of similar importance as when using VOG for HIT. Nevertheless, the VOG and EOG recorded eye movement traces showed a close correlation, which suggests that in this study no major or very similar electrode or goggle movement artifacts were present in the eye movements recorded by both techniques. Moreover, using a separate head movement sensor (e.g. on a bite bar) will allow to include all impulses independent of the rejection algorithms of any oculography system. It will be implemented in the follow-up studies aimed to analyze the vertical (LARP and RALP) HITs.

### Future perspectives

This study investigated the feasibility of EOG to accurately detect eye movements during the horizontal HIT, which was confirmed based on the close correlation between EOG and VOG recorded eye velocities. This could pave the way for the development of an EOG-based HIT system, incorporating a motion sensor (e.g., mounted on a bite board), and determining its accuracy for clinical use. Furthermore, this study could be extended to vertical HITs, in which especially the use of VOG can be challenging due to pupil detection difficulties [i.e., the pupil can be covered by the eyelids, (35)], whereas EOG might be easier to apply with sufficient accuracy (36).

Using a head-fixed motion sensor together with a reliable synchronization pulse between the two oculography systems (e.g., a special eye movement pattern added in the beginning and/or at the end of the recording) might give an insight in slippage sensitivities of both eye movement recording techniques. This could also facilitate evaluating the delays between head and eye traces, and therefore, estimate possible gain calculation errors due to goggle slippage (26).

EOG and VOG methods can be compared in terms of the signal-to-noise ratio and accuracy or resolution using both raw and unfiltered traces. The resolution for EOG is believed to be close to 0.5°, whereas for VOG, it is quantified at about 0.01° (8). However, these average numbers can substantially vary from patient to patient and very strongly depend on the typical features of pupil detection vs. corneoretinal potential detection.

## Conclusion

To our knowledge, this is the first description of using the EOG technique to record eye movements during head impulse testing. EOG and VOG obtained eye movement traces correlate well with each other during horizontal head impulse testing. In addition, EOG did not show significant VOR gain asymmetry in healthy individuals, in contrast to VOG. These findings indicate that EOG might potentially be applicable as an alternative to VOG for collecting eye movement responses during head impulse testing.

## Data availability statement

The raw data supporting the conclusions of this article will be made available by the authors, without undue reservation.

## Ethics statement

The studies involving human participants were reviewed and approved by local Ethical Committee of Tomsk State University. The patients/participants provided their written informed consent to participate in this study.

## Author contributions

MP and VZ collected the data. MP, VZ, and DS conducted the analysis and wrote the manuscript. VD, HK, and RB supervised the study and edited the manuscript. All authors contributed to the article and approved the submitted version.

## Conflict of interest

The authors declare that the research was conducted in the absence of any commercial or financial relationships that could be construed as a potential conflict of interest.

## Publisher's note

All claims expressed in this article are solely those of the authors and do not necessarily represent those of their affiliated organizations, or those of the publisher, the editors and the reviewers. Any product that may be evaluated in this article, or claim that may be made by its manufacturer, is not guaranteed or endorsed by the publisher.
